# Mechanism and Performance Control Methods of Sulfate Attack on Concrete: A Review

**DOI:** 10.3390/ma17194836

**Published:** 2024-09-30

**Authors:** Chuanchuan Zhang, Julun Li, Miao Yu, Yue Lu, Shizhong Liu

**Affiliations:** 1 School of Vehicle and Transportation Engineering, Taiyuan University of Science and Technology, Taiyuan 030024, China; zccmech2012@163.com (C.Z.); shizhong_liu@tyust.edu.cn (S.L.); 2 Department of Structural Engineering, Tongji University, Shanghai 200092, China; yumiao891@tongji.edu.cn (M.Y.); y_lu@tongji.edu.cn (Y.L.)

**Keywords:** cement-based materials, sulfate attack, corrosion mechanism, environmental factors, material factors, modification

## Abstract

For concrete structures in marine or groundwater environments, sulfate attack is a major factor contributing to the degradation of concrete performance. This paper analyzes the existing literature on the chemical reactions and physical crystallization effects of sulfate attack on cement-based materials, summarizing the degradation mechanisms of corroded concrete. Experiments have been conducted to study the performance evolution of concrete under sulfate attack, considering both external environmental factors and internal factors of the cement-based materials. External environmental factors, such as the temperature, humidity, concentration, and type of sulfate solutions, wet-dry cycles, freeze-thaw cycles, chloride coupling effects, and stray currents significantly impact sulfate attack on concrete. Internal factors, including internal sources of corrosion, the chemical composition of the cement, water-cement ratio, and the content of C-S-H gel and Ca(OH)_2_, influence the density and sulfate resistance of the cement-based materials. Additionally, five typical methods for enhancing the sulfate resistance of concrete are summarized. Finally, the paper identifies current challenges in the study of corroded concrete and proposes directions for future research.

## 1. Introduction

Sulfate attack is a significant durability issue for cement-based materials, affecting structures and components such as dams, concrete pavements, bridge piers, concrete piles, embedded concrete foundations, and slabs, in addition to highway or railway tunnels [1]. Due to its porous structure, concrete permits the ingress of sulfates into its matrix via pore solution driven by hydrostatic pressure [2]. Upon the infiltration of sulfate ions into the concrete interior, they engage in reactions with hydration products, resulting in the formation of expansive compounds, such as ettringite [3]. This process induces a range of degradation phenomena in the concrete, characterized by swelling, cracking, spalling, reduced strength, and a deterioration in overall quality [4,5]. These degradation phenomena ultimately lead to the failure of concrete structures, significantly limiting their suitability for use in environments such as coastal saline soils, inland salt lakes, and saline-alkali terrains [2]. The factors contributing to concrete damage due to sulfate attack are multifaceted and markedly detrimental. To ensure that concrete infrastructures meet their functional expectations throughout their entire service life, an increasing number of researchers are investigating the sulfate resistance of concrete under diverse environmental conditions [6,7,8].

In practical engineering scenarios, the reduction of concrete durability seldom stems from a solitary influence, but rather from the synergistic impact of several factors [9]. Concrete structures situated in areas with fluctuating water levels, splash zones, and tidal variations are exposed to the compounded effects of alternating dry and wet cycles in conjunction with sulfate attack, leading to exacerbated damage [10,11]. Specifically, in the sulfate attack environments of cold regions, where winter temperatures persist below freezing for extended periods, concrete infrastructures face not only sulfate attack, but also the harmful effects of freeze-thaw cycles, thus further complicating the deterioration processes of concrete [12,13].

However, the current literature often focuses on isolated scenarios, such as the effects of sulfate attack alone or the impact of individual salts in controlled environments. This narrow focus fails to address the complexity of real-world conditions, where concrete structures are frequently exposed to a combination of aggressive agents and varying environmental stressors. Moreover, the interactions between different types of salts, such as sodium sulfate and magnesium sulfate, and their combined effects on concrete durability have not been thoroughly explored. Each type of salt can induce different chemical reactions and physical changes within the concrete matrix, resulting in unique patterns of damage. Therefore, a more comprehensive and detailed summary of sulfate resistance in concrete is necessary; one that considers the multifaceted and synergistic impacts of various environmental factors and types of salts. This would provide a more realistic and applicable understanding of concrete durability in diverse and challenging conditions, guiding better design and maintenance practices for concrete structures in sulfate-rich environments.

To fill the addressed gap, this review summarizes the deterioration mechanisms and patterns of concrete under the coupled effect of sulfate attack and environmental factors. It offers a comprehensive overview of the current state of research, including experimental and theoretical models that examine the mechanical properties of concrete materials influenced by sulfate attack. This includes the interplay with environmental temperature and humidity, the types and concentrations of sulfates, freeze-thaw cycles, wet-dry cycles, chlorine salt attack, and stray current. In addition, the effects of material factors (such as internal erosion source, cement chemical composition, water-cement ratio, and geopolymers) on sulfate attack concrete are also summarized. The research status of improving concrete resistance to sulfate attack is further analyzed. Ultimately, the review identifies significant gaps in the existing research on concrete exposed to sulfate attack and proposes directions for future investigations.

Through analysis of existing studies, this study explores the physical processes and chemical reactions involved in sulfate attack on concrete, categorizing various hypotheses proposed by scholars. This review elucidates the mechanisms by which sulfate attack affects the integrity of concrete structures, including the reactions between sulfates and cement hydration products that lead to concrete performance degradation. It offers a comprehensive evaluation of the factors influencing sulfate attack on concrete and outlines the primary methods for regulating performance. This work aims to provide researchers and engineers with a clear reference framework.

## 2. Mechanism of Concrete Deterioration under Sulfate Attack Conditions

The deterioration and damage of concrete structures caused by sulfate attack arise from the interactions between corrosive ions and the hydration products in concrete, resulting in the formation of expansive compounds. This process initiates cracking and spalling in concrete, facilitating deeper penetration of corrosive agents, which accelerates the corrosion of reinforcing bars, further compromising the structural integrity and reducing its load-bearing capacity. Therefore, investigation into the deterioration and damage of concrete structures due to sulfate attack necessitates a foundational understanding of the concrete’s degradation mechanisms by sulfates. Typically, the mechanisms of sulfate attack on concrete are categorized into two distinct types: chemical attack, which involves the chemical alteration of concrete components; and physical attack, which results from physical changes induced by the presence of sulfates [14].

### 2.1. Physical Attack

#### 2.1.1. Solid Volume Expansion

It is proposed that the primary cause of concrete deterioration due to sulfate attack is the change in the volumetric expansion of the solid phase. Specifically, this involves an increase in the volume of sulfate crystals when they transition from their anhydrous form to their hydrated form [15,16,17]. Among the sulfates, sodium sulfate (Na_2_SO_4_) is identified as the most impactful in terms of solid phase volume expansion, exhibiting a 311% increase in volume upon hydration. However, this theory does not account for the water consumption throughout the entire sulfate attack process on concrete. When considering the volume of water involved in the crystallization of sulfates, the net volume of the system actually decreases. For instance, the crystallization transformation of anhydrous sodium sulfate (Na_2_SO_4_) into its decahydrate form (Na_2_SO_4_·10H_2_O) [18], as outlined in Equation (1), results in a 315% increase in solid volume, but the total volume of the products decreases by 5.6% compared to the total volume of the reactants [19]. It should be noted that, though this reaction is fundamentally chemical, the sulfate does not chemically interact with the internal components of the concrete. Instead, it combines with free water to form larger salt crystals. The adverse impact on concrete performance is due to the force exerted by Na_2_SO_4_·10H_2_O crystals on the pore walls within the matrix. Therefore, this phenomenon is considered a form of physical decay in cement-based materials in this paper.

(1) $${Na}_{2}{SO}_{4}+10H_{2}O\Leftrightarrow {Na}_{2}{SO}_{4}\cdot 10H_{2}O$$

However, the decisive condition for the occurrence of the Na_2_SO_4_ crystallization reaction is the ability of the solution to reach supersaturation. Supersaturated solutions can be generated through cooling, evaporation, and wet-dry cycles. Therefore, the reaction shown in Equation (1) has limited correlation with sodium chloride (NaCl). Since NaCl does not form a hydrated solid, this theory does not adequately explain the damage mechanism associated with NaCl erosion [20].

#### 2.1.2. Pressure of Crystalline Water

This theory is predicated on three fundamental assumptions: salts within the pores remain stationary, salts maintain contact with the external environment, and the external relative humidity exceeds the equilibrium humidity required for the transition between two crystalline salt forms. Under these conditions, the theory posits that anhydrous salts absorbing moisture and undergoing solid-state hydration can exert pressure on the surrounding pore walls, thereby causing damage to porous materials [21].

However, this theory does not take into consideration the distinct crystal structures between hydrated and anhydrous salts, suggesting that a simple hydration reaction is insufficient to alter the crystal structure. Folliard and Sandberg [22] have demonstrated through analysis of solution concentrations that solid sodium sulfate does not merely hydrate. Instead, sodium sulfate crystals continuously dissolve, leading to a supersaturation of the sodium sulfate solution. This supersaturation then results in the crystallization of anhydrous sodium sulfate crystals.

#### 2.1.3. Pressure of Crystal Expansion

This theory suggests that solutes in a supersaturated solution carry greater potential energy than those in a saturated solution of the same composition. As solutes precipitate from the supersaturated state, this additional potential energy provides the necessary force to overcome any external pressures that may inhibit crystallization [20].

This pressure is applied to the internal pore walls of concrete, resulting in structural degradation within the material. Correns [23] introduced a method to calculate the pressure exerted by salt crystallization, as outlined in Equation (2). From this formula, it can be inferred that salt crystals will only form in a supersaturated solution. Variations in the concentration of sulfates residing in the pores may arise as a consequence of cyclic dry-wet phases, coupled with the process of moisture evaporation on the concrete surface, thereby impacting the crystallization parameters.

(2) $$P=\frac{RT}{V}ln\left(\frac{C}{Cs}\right)$$

where *P* represents the pressure generated by the growth of the crystal, *R* represents the ideal gas constant, *T* represents the absolute temperature, *V* represents the solid volume of the salt, *C* represents the concentration of the solution, and *C_s_* represents the supersaturation concentration of the solution.

Distinct from the theory of pressure of hydrated salt, the crystallization pressure theory posits that the principal mechanism behind the generation of crystallization pressure is the expansion of crystals post-nucleation, which in turn contributes to the disintegration of concrete [24].

### 2.2. Chemical Attack

The chemical reactions involved in sulfate attack on concrete mainly include the formation of ettringite (Aft, 3CaO·Al_2_O_3_·3CaSO_4_·32H_2_O) and gypsum (CaSO_4_·2H_2_O) [19]. The reaction processes are detailed in Equations (3)–(5) [25,26]. Ettringite is one of the early hydration products of Portland cement, observable within 10 min of hydration initiation [27]. During the initial hours of Portland cement paste hydration, ettringite often forms in needle-like rod shapes. Typically, the gypsum content in Portland cement is around 5%, while the C_3_A content generally exceeds 5%. Consequently, some ettringite converts to monosulfate (AFm, 3CaO·Al_2_O_3_·3CaSO_4_·xH_2_O) due to gypsum depletion. When the C_3_A content is above 8%, both monosulfate and hydrated calcium aluminate can form. This early-forming ettringite is harmless to concrete, as it forms before the cement fully hardens and may decompose at later stages.

In the later stages of hydration, when hardened cement paste comes into contact with a sulfate-containing environment, SO_4_^2−^ ions react with AFm and hydrated calcium aluminate in the cement to form ettringite. This secondary ettringite is the main chemical reaction product associated with expansion, cracking, and spalling, as illustrated in reactions (4–6) [25]. The primary cause of concrete deterioration is the intrusion of SO_4_^2−^ ions into the concrete pores, where they react with hydration products to form ettringite. The formation of ettringite significantly increases the solid phase volume to approximately eight times that of tricalcium aluminate (3CaO·Al_2_O_3_). Due to the needle-like nature of ettringite crystals, substantial stress is generated, leading to macroscopic manifestations such as concrete expansion and cracking, with a few coarse cracks appearing on the concrete surface.

(3) $$Ca{\left(OH\right)}_{2}+{Na}_{2}{SO}_{4}+2H_{2}O\to Ca{SO}_{4}\cdot 2H_{2}O+2NaOH$$

(4) $$2(Ca{SO}_{4}\cdot 2H_{2}O)+3CaO\cdot {Al}_{2}O_{3}\cdot Ca{SO}_{4}\cdot 12H_{2}O+16H_{2}O\;\to 3CaO\cdot {Al}_{2}O_{3}\cdot 3Ca{SO}_{4}\cdot 32H_{2}O$$

(5) $$3\left(Ca{SO}_{4}\cdot 2H_{2}O\right)+3CaO\cdot {Al}_{2}O_{3}+26H_{2}O\to 3CaO\cdot {Al}_{2}O_{3}\cdot 3Ca{SO}_{4}\cdot 32H_{2}O$$

Therefore, ettringite can be categorized into two types: one type is a hydration product formed during the early hydration of cement, and the other type is expansive ettringite formed by the reaction between hydration products in the hardened cement paste and SO_4_^2−^ at a later stage. Researchers [28] have distinguished ettringite into two types using different methods: (i) long prismatic crystals with sizes ranging from 10 µm to 100 µm, formed at lower OH^−^ concentrations, exhibiting high strength without expansion; and (ii) rod-shaped crystals with sizes ranging from 1 µm to 2 µm, formed at higher OH^−^ concentrations, which are classified as destructive ettringite.

## 3. Influence of External Environmental Factors on the Sulfate Attack Resistance of Concrete

The environments surrounding concrete structures are diverse and variable, including a wide range of conditions. Factors such as temperature and humidity, as well as the concentration and types of sulfate solutions, vary considerably. These environmental variables significantly influence the susceptibility and intensity of sulfate attacks on concrete.

### 3.1. Environmental Temperature

**Figure 1:**
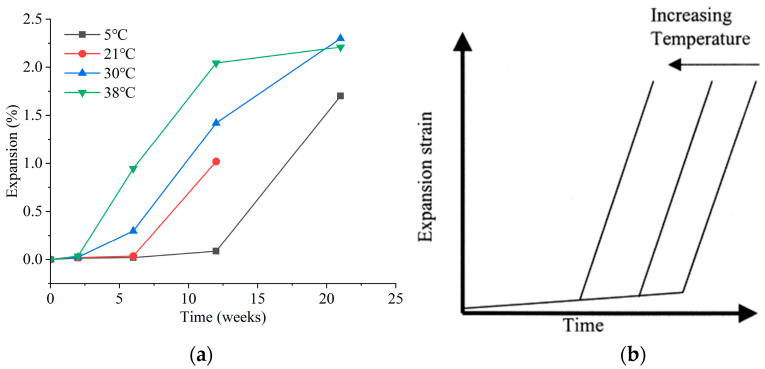
Effect of temperature variations on the corrosion patterns of mortar by sulfates. (**a**) Expansion patterns of mortar corroded by sodium sulfate at different temperatures; (**b**) schematic illustration of the expansion patterns of mortar corroded by sodium sulfate as influenced by temperature [32].

Wu [29] investigated the effect of temperature variations on the damage of cement mortar by magnesium sulfate solutions. The results indicated a complex pattern of sulfate attack on cement mortar, characterized by an initial phase of enhanced performance, followed by a deterioration phase. Notably, cement mortar subjected to corrosion at low temperatures exhibited a higher susceptibility to damage compared to that at normal temperatures. However, the effect of temperature variations on cement mortar with a low water-cement ratio was found to be minimal. Chen et al. [30], on the other hand, argue that temperature not only impacts the rate of sulfate attack on concrete, but also affects the types of reaction products formed, thereby altering the concrete’s mechanical properties. Their findings indicate that at temperatures of 20 °C and 35 °C, the penetration depth of SO_4_^2−^ into concrete progressively increases, as does the concentration of SO_4_^2−^ at equivalent depths of corrosion. Conversely, at an environmental temperature of −15 °C, the diffusion speed of SO_4_^2−^ is significantly reduced. After 480 days of exposure, the depth of diffusion was observed to be only between 3 to 5 mm [31]. Santhanam et al. [32] conducted experimental investigations into the effects of sulfate attack on cement mortar across four distinct temperature settings. The results demonstrated that the volumetric expansion of mortar subjected to sulfate attack follows a two-stage pattern. The increase in temperature shortened the duration of the first stage of expansion, but did not cause significant changes to the pattern of the second stage of expansion, as shown in Figure 1. However, the ettringite produced during corrosion began to decompose when the temperature reached 70 °C, indicating a change in the mechanism of sulfate attack. The research conducted by Fang et al. [33] also indicates the existence of a critical temperature threshold in sulfate attack. When the temperature is below this critical threshold, an increase in temperature aggravates the decline of the concrete’s mechanical properties. Conversely, when the temperature exceeds the critical point, the accelerated decomposition of delayed ettringite formation leads to a slowdown in the degradation of the concrete’s mechanical performance [28].

### 3.2. Environmental Humidity

Variations in the humidity of the concrete’s environment can lead to different sulfate corrosion mechanisms and the formation of different crystalline reaction products. For instance, in the case of concrete corrosion by a sodium sulfate solution [34], when the environmental temperature is maintained at 20 °C and the relative humidity is at 45%, the predominant corrosion product is anhydrous sodium sulfate (Na_2_SO_4_) crystals. However, at a relative humidity of 85%, the primary product formed is Na_2_SO_4_·10H_2_O crystals. Therefore, the formation of Na_2_SO_4_ crystals necessitates a lower humidity environment, that is, a need for substantial water evaporation from the surface of cementitious materials, which accelerates the rate of sodium sulfate solution transport within the internal pores. As mentioned, there are several potential sources of sodium (Na). Firstly, the raw materials used in concrete preparation may inherently contain Na_2_SO_4_. For example, certain researchers are currently developing seawater sea-sand concrete. Secondly, Na_2_SO_4_ from the surrounding environment can infiltrate the concrete. In some regions, groundwater, saline soils, and water in rock fractures may all contain Na_2_SO_4_. To investigate the impact of different humidity conditions on concrete subjected to sulfate attack, Nehdi et al. [35] conducted experiments under two differerent humidity settings: one with the relative humidity consistently controlled at approximately 32 ± 3%, and another with the relative humidity varying between a minimum of 32 ± 3% and a maximum exceeding 95%, cycling continuously between these values. Their findings revealed that under steady relative humidity, the mortar exposed to sulfates produced only a limited quantity of crystals on the solution surface. In contrast, a cycling humidity environment led to the formation of a substantial amount of white crystals on the mortar’s surface. However, their study did not extend to comparing other levels of humidity or varying the cycling intervals within these two humidity conditions.

### 3.3. Concentration of Sulfate Solution

The mechanism of sulfate corrosion is closely related to the solution concentration, and the products generated by sulfate corrosion of concrete are different for different SO_4_^2−^ concentrations [36]. For sodium sulfate, environments with low SO_4_^2−^ concentration (below 1000 mg/L) predominantly yield gypsum as the main reaction product. In contrast, in high SO_4_^2−^ concentration environments (between 1000 to 8000 mg/L), both gypsum and ettringite are observed. For magnesium sulfate, environments with low SO_4_^2−^ concentration (below 4000 mg/L) predominantly yield gypsum as the main reaction product. In higher SO_4_^2−^ concentration environments (between 1000 to 7500 mg/L), both gypsum and ettringite are observed. The corrosion of magnesium ions will dominate when the SO_4_^2−^ concentration is above 7500 mg/L [37].

Ou et al. [38] examined the effects of sulfate solution corrosion on relative mass loss and the strength corrosion resistance coefficient of concrete at three different concentrations (Table 1). It was found that as the concentration of the solution increased, the relative mass loss of the concrete was greater, and the strength corrosion resistance coefficient was lower, demonstrating a strong linear relationship between these two variables. Fang et al. [33] investigated the temporal variations in the flexural and compressive corrosion resistance coefficients of concrete when exposed to sulfate solutions of varying concentrations. The results highlighted that both the flexural and compressive corrosion resistance coefficients experienced phases of increase and decrease. Below a certain sulfate concentration, the rate of sulfate attack accelerated with an increase in concentration. However, when the concentration exceeded this critical value, the rate of corrosion slowed down, and lower sulfate solution concentrations resulted in longer durations of the increasing phase. Fan et al. [39] performed uniaxial compression tests on concrete subjected to sodium sulfate solution corrosion at four different concentrations over a period of 210 days and found an upward trend in the number of microcracks within the concrete with increasing sodium sulfate concentration, facilitating quicker penetration of sulfate ions. This resulted in the accumulation and expansion of corrosion products, adversely affecting the concrete’s mechanical properties. Chen et al. [40] characterized the internal damage of concrete samples corroded by Na_2_SO_4_ solutions of differing concentrations using changes in ultrasonic pulse velocity. Their findings indicated that higher concentrations led to more extensive damage to the concrete structure [41].

### 3.4. Types of Sulfates

Currently, the impact of sulfate types on concrete corrosion is primarily studied based on the cations present in the salt solutions, categorized into soluble ions, such as Na^+^ and K^+^, and insoluble cations (Mg^2+^). These are the most common types of cations in the environment and are extensively studied in sulfate attack research. Different sulfate types lead to different corrosion mechanisms. The primary products of MgSO_4_ attack on concrete are gypsum and brucite [3,42]. In contrast, Na_2_SO_4_ attack primarily produces ettringite, as illustrated in the accompanying XRD pattern. Moreover, magnesium sulfate can react with ettringite to form magnesium silicate hydrate (M-S-H), which lacks binding capability.

**Figure 2:**
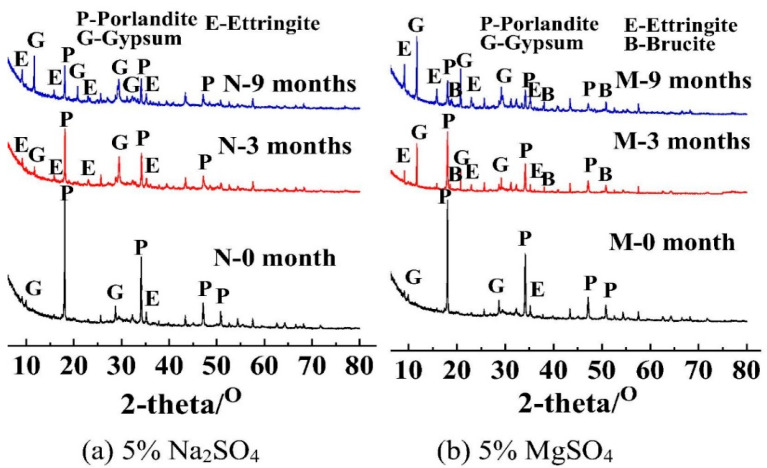
The XRD patterns of specimens immersed in (**a**) 5% Na_2_SO_4_ solution and (**b**) 5% MgSO_4_ solution [44].

In addition to the formation of gypsum and ettringite from the reaction with SO_4_^2−^, MgSO_4_ also involves Mg^2+^, which reacts to form insoluble Mg(OH)_2_, lowering the pH of the solution and causing severe decalcification reactions (pH < 11.4). This leads to the decomposition of C-S-H gel in concrete [43]. Furthermore, Mg^2+^ reacts with the hydration products in concrete to form non-cementitious hydrated magnesium silicate, thus accelerating the dissolution of hydration products and the formation of expansive products, and resulting in the softening of the concrete’s surface layer. The XRD patterns of specimens immersed in different solutions are shown below in Figure 2.

**Figure 3:**
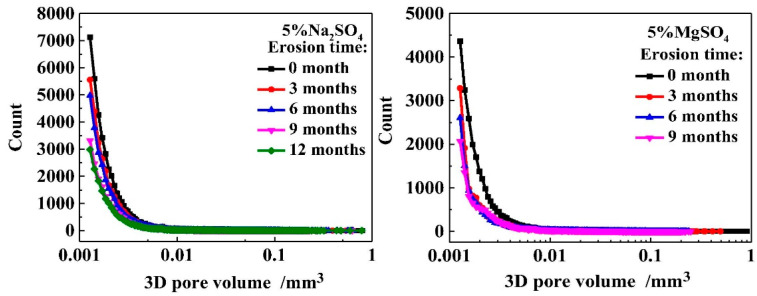
The porosity of the specimens immersed in 5% Na_2_SO_4_ solution and 5% MgSO_4_ solution [44].

The development of porosity in samples soaked in different sulfate solutions is illustrated in Figure 3 [44]. Yang et al.’s [44] research shows that the porosity of cement paste samples soaked in 5 wt% Na_2_SO_4_ solution for 9 months decreased from an initial 0.64% to 0.4%. In contrast, the porosity of samples soaked in an MgSO_4_ solution of the same concentration for the same period decreased from an initial 0.67% to 0.37%. This indicates that the internal pores of cement paste subjected to magnesium sulfate attack were more thoroughly filled. Furthermore, the number of small pores in the range of 0.001–0.01 mm^3^ in samples corroded by magnesium sulfate solution decreased more significantly.

**Figure 4:**
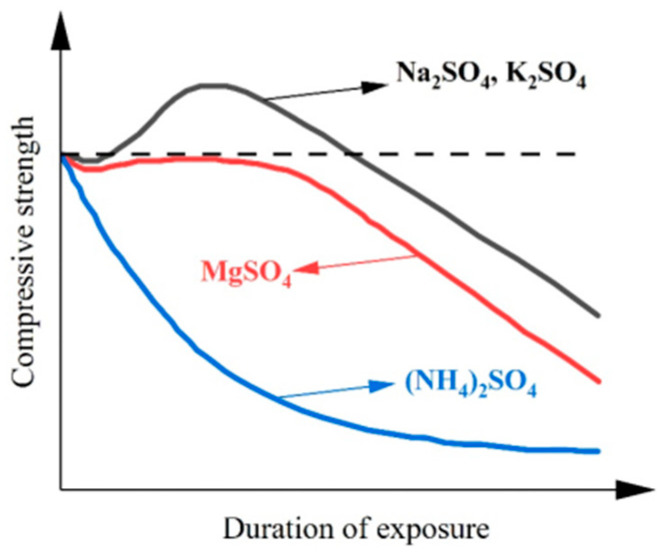
Deterioration of compressive strength by different types of sulfates [46].

The type of sulfate solution affects the degree of concrete deterioration differently. Compared to Na_2_SO_4_ solutions, concrete exposed to MgSO_4_ solutions exhibits greater thickness of the damaged layer, more pronounced deterioration in compressive strength, and more severe damage [45]. Xiong et al. [46] categorized the cross-section of cement paste specimens exposed to sulfate attack into three zones, the corrosion zone, the reaction zone, and the intact zone, and concluded that within the reactive zone, the sulfate type had a significant effect on the microhardness of the cement mortar [47]. The rate of hardness reduction follows this pattern: Na_2_SO_4_ ≈ K_2_SO_4_ > MgSO_4_ > (NH_4_)_2_SO_4_, as illustrated in Figure 4.

### 3.5. Wet-Dry Cycles

Sulfate environments, such as coastal lakes, are often characterised by wave-splash and tidal phenomena, making concrete structures highly susceptible to wet-dry cycling mechanisms [48]. In sulfate environments, the primary distinction between wet-dry cycling and fully submerged conditions lies in the fact that during dry periods, the evaporation of internal moisture in concrete rapidly increases the concentration of Na_2_SO_4_ solutions, leading to the crystallization of Na_2_SO_4_. Under the combined effects of sulfate attack and wet-dry cycling, the microstructure of concrete undergoes a process where corrosion products continuously accumulate at the interface between the cement paste and aggregate within the concrete’s pores. This is accompanied by the destruction of sulfate crystals and the generation of microcracks in the concrete in the corrosion zone. The microcracks continuously expand and connect, which ultimately leads to the process of deterioration and destruction of the concrete.

**Figure 5:**
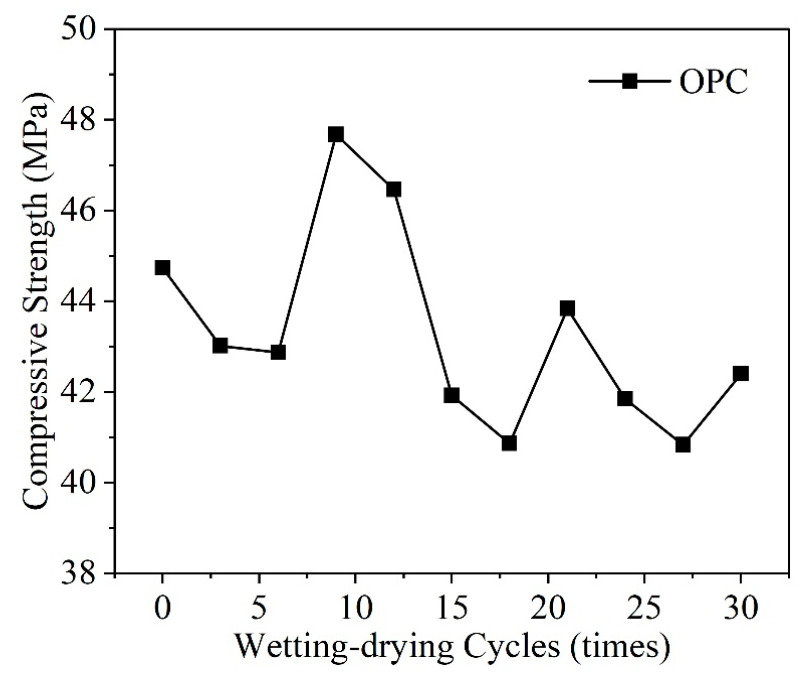
Relationship between compressive strength of concrete and the number of wet-dry cycles [49].

The wet-dry cycling mechanism accelerates the sulfate attack reaction, and as the number of wet-dry cycles increases, the by-products accumulate in the concrete pores, generating crystallization pressure and exacerbating the damage caused by sulfate corrosion to the concrete [49]. Prior to the sixth wet-dry cycle, concrete damage primarily results from Na_2_SO_4_ corrosion and shrinkage during the drying phase. However, at this stage, the rate of the corrosion reaction is slow, and the expansive crystals are not sufficient to damage the concrete. Figure 5 presents the relationship between the compressive strength of concrete and the number of wet-dry cycles. Li et al. [50] developed the diffusion model for SO_4_^2−^ under wet-dry cycling conditions, indicating that the wet-dry mechanism accelerates the intrusion of SO_4_^2−^, thereby exacerbating the degradation of concrete strength. Xia [51] analyzed the impact of the number of wet-dry cycles on the corrosion of cement soil by sulfate solutions, finding that the rate of mass loss in cement soil increases with the number of cycles, reaching a maximum after 28 cycles; meanwhile, the unconfined compressive strength first increases then decreases, peaking after seven cycles. Unlike sulfate-corroded cement soil, the mass and density loss rates of concrete tend to increase with the number of wet-dry cycles, showing a pattern of initially rapid and then slower increases, without reaching a turning point [52].

The research by Han et al. [53] indicates that the coupled effect of sulfate attack and wet-dry cycling does not affect the initial linear portion of the stress–strain curve of high ductility cement-based materials, but significantly impacts the nonlinear rising and stress declining segments. Furthermore, the compressive strength significantly increases with the number of corrosion cycles and wet-dry cycles, reaching its peak after four cycles. In contrast, Wang et al. [54] argues that the coupling of wet-dry cycling with sulfate attack rapidly deteriorates the mechanical properties of concrete. Yang [55] utilized the relative dynamic modulus of elasticity as an evaluative index for the cumulative damage model of cement-based materials and fitted a cumulative damage prediction model according to the damage read evolution law, shown in Equation (6). The results show that the damage rate of concrete increases with the number of wet and dry cycles, and the greater the water–cement ratio, the greater the extremely incremental damage rate.

(6) $$D_{t}=at^{2}+bt+c$$

where $D_{t}$ is the degree of damage, *t* is the number of wet and dry cycles, and *a, b*, and *c* are coefficients associated with the test.

### 3.6. Freeze-Thaw Cycle

Among the various mechanisms of freeze-thaw damage in concrete, the hydrostatic pressure mechanism and the osmotic pressure mechanism are the most representative. During the freeze-thaw process in concrete, the expansion of ice forming from capillary water generates hydrostatic pressure, while osmotic pressure arises from supercooled gel water migrating towards the ice interface. This results in the formation of fine cracks in the frozen zones of saturated concrete [56,57,58]. The continuous expansion of microcracks in the frozen zones leads to spalling, and the freeze-thaw cycles also affect macroscopic properties such as the mass, relative dynamic modulus of elasticity, and compressive strength of concrete [54,59].

With an increase in the number of freeze-thaw cycles, the expansive stress caused by the byproducts of sulfate attack in concrete, combined with the effects of freezing expansion, further exacerbates the internal deterioration of the concrete [60], leading to a rapid decrease in peak stress [61], a gradual reduction in elastic modulus, and an increase in peak strain [62]. Li [63] studied the durability of concrete with different water–cement ratios under the coupled effects of freeze-thaw cycles and sulfate solution attack. The results showed that a higher water–cement ratio in concrete corresponds to greater mass loss and poorer resistance to corrosion. Furthermore, other researchers [64] have also introduced the damage degree and developed a freeze-thaw damage model for recycled concrete in sulfate corrosion environments based on damage mechanics, as shown in Equation (7). Using this model to predict the durability life of recycled concrete, results indicate that the freeze-thaw durability life of concrete in 3%, 5%, and 10% Na_2_SO_4_ solutions decreases by 15.8%, 24.2%, and 17.2%, respectively, compared to in pure water.

(7) $$D_{n}=An^{2}+Bn$$

where *D_n_* is the degree of damage, *n* is the number of freeze-thaw cycles, and *A* and *B* are coefficients to be determined with respect to the concentration of the sulfate solution.

### 3.7. Chloride Coupling

**Figure 6:**
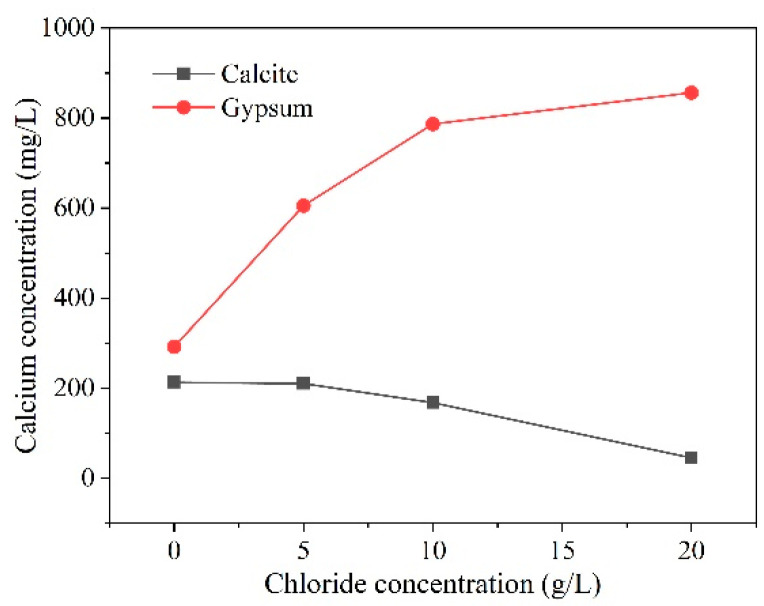
Effect of chloride ions on the solubility of calcite and gypsum [65].

In practical engineering environments, chlorides often coexist with sulfates, acting in concert to damage concrete structures. Unlike sulfate attack, the primary reaction product of chloride attack on concrete is Friedel’s salt, which is less expansive compared to ettringite. Furthermore, the presence of chloride ions influences the solubility of calcite and gypsum, with this effect being highly sensitive to concentration changes. Specifically, the solubility of calcite decreases as chloride ion concentration increases, whereas the solubility of gypsum increases with higher chloride ion concentrations [65], as illustrated in Figure 6 below.

**Figure 7:**
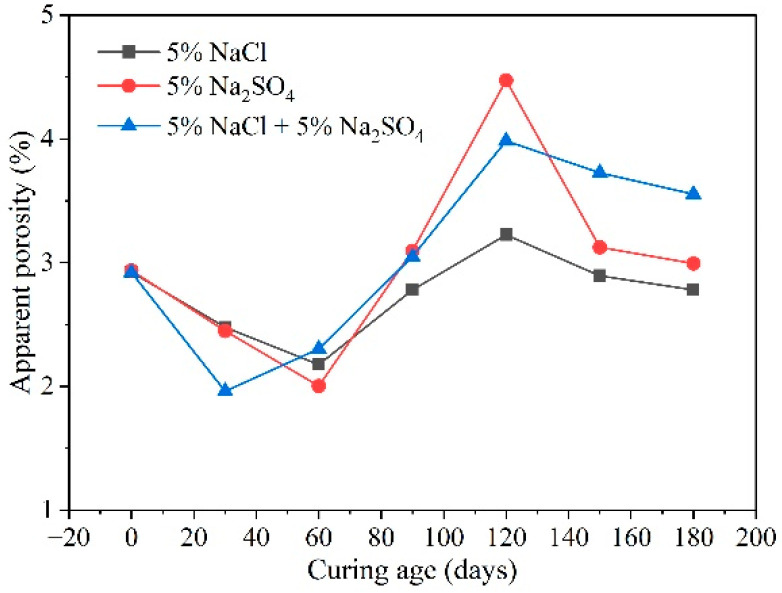
Apparent porosity of mortar in different solutions [66].

Chloride ions also influence porosity. Generally, the change in porosity over time can be divided into three stages: an initial decrease, followed by an increase, and a subsequent decrease, as shown in Figure 7 [66]. The combined effect of chloride and sulfate salts causes the porosity of mortar to increase earlier than when either chloride or sulfate salts are present alone. This is because the chloride salts, which first penetrate the specimen, react with minerals like C_3_A to form Friedel’s salt. This reaction fills large pores, refines pore sizes, and limits the formation of expansive corrosion products such as ettringite after sulfate ion penetration.

Currently, there is no universal consensus on the impact of chlorides on concrete subjected to sulfate attack, and primarily, two contrasting conclusions have been drawn.

The first concludes that chloride salts exacerbate the corrosive effects of sulfates on concrete. The presence of NaCl not only exacerbates the corrosion of concrete by Na_2_SO_4_, but also intensifies the damage caused by MgSO_4_ under low temperatures (around 5 °C) [67]. Due to the influence of chloride on the solubility of calcite and gypsum—a phenomenon that exhibits notable sensitivity to concentration levels—a chloride concentration of 0.5% can accelerate the reaction of sulfate ions in the pore solution to form ettringite, thereby leading to mortar deterioration [65]. It has also been demonstrated that increasing the sulfate concentration in a 15.7% NaCl solution from 0.55% to 2.1% doubles the corrosion rate of steel reinforcement [68], thereby increasing internal stress within the concrete and exacerbating damage.

**Figure 8:**
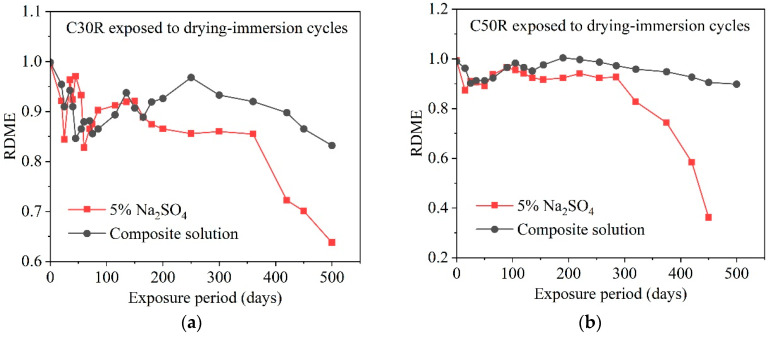
Relative dynamic modulus of elasticity development pattern of concrete in single and double salt solutions [71]. RDME of C30R (**a**) and C50R (**b**) concrete stored in 5% Na_2_SO_4_ and in composite solution of 3.5% NaCl and 5% Na_2_SO_4_.

The second concludes that the presence of chlorides has been found to alleviate the effects of sulfate attack on concrete [69,70]. In concrete exposed to a 5% Na_2_SO_4_ solution and subjected to wet-dry cycles over 500 days, the damage process can be divided into four stages based on the relative dynamic modulus of elasticity (RDME): (I) initial decline, (II) linear increase, (III) slow decline, and (IV) accelerated destruction. Figure 8 presents the relative dynamic modulus of elasticity development pattern of concrete in single and double salt solutions. It can be seen that, for the composite solution, only the first three stages occur within the 500-day accelerated corrosion period [71]. Compared to concrete immersed in a solution of 3.5% NaCl and 5% Na_2_SO_4_, the damage is more severe in concrete soaked in 5% Na_2_SO_4_ alone. This indicates that the presence of chlorides in the composite solution extends the duration of each phase, and the deterioration caused by SO_4_^2−^ is delayed. Some researchers [72] believe that chlorides reduce the supersaturation of ettringite and explain the mitigating mechanism of chlorides on pore pressure and thus sulfate attack from this perspective.

### 3.8. Stray Current

Currently, research on the degradation processes, mechanisms, and models of cementitious materials under the coupled action of stray currents and sulfate is still insufficient, with only a few scholars, such as Lorente and Wang, conducting related studies [73,74,75].

Lorente et al. [75] compared the effects of electric field forces induced by external electric fields and concentration gradients on the transport of sulfate ions in cementitious materials. Additionally, the impact of different cations (Na^+^, Mg^2+^) on the transport of SO_4_^2−^ within cementitious materials and their mechanical properties were studied in the presence of an external electric field. Experimental results showed that the migration caused by external electric fields significantly accelerated the transport of sulfate ions from the external solution into the specimens. However, when the cations in the sulfate solution were Mg^2+^, the concentration of SO_4_^2−^ in the specimens was higher than under conditions with Na^+^ as the cation. When there was no external electric field, the concentration of sulfate ions in the specimens was reversed compared to the aforementioned conditions, the reasons for which require further investigation. The research listed the migration equation for sulfate ions under the influence of an external electric field as shown in Equation (8). Lorente theoretically estimated the time required for SO_4_^2−^ to pass through a concrete specimen when the diffusion coefficient D is at the order of 10^−12^ and the external field voltage is 50 V. It was found that SO_4_^2−^ could pass through a concrete specimen with a thickness of 11 cm in just 6 days, indicating that the external electric field has a significant accelerating effect on the transport of SO_4_^2−^ in the specimen, and it is more significant than the free diffusion process caused by concentration gradients. However, this diffusion formula did not consider the impact of chemical reactions on the transport of SO_4_^2−^ in concrete, nor did it take into account the effects of sulfate-induced degradation (such as cracking) on the transport process.

(8) $$\frac{{\partial C}_{T}}{\partial t}=D\left(\frac{\partial^{2}c}{\partial x^{2}}+z\frac{F}{RT}\cdot \frac{\partial c}{\partial x}\cdot \frac{\partial \varphi }{\partial x}\right)$$

where ${\partial C}_{T}$ denotes the total concentration of SO_4_^2−^ (mol/m^3^), *c* represents the concentration of free SO_4_^2−^ in the pore solution (mol/m^3^), *D* is the diffusion coefficient for SO_4_^2−^ (m^2^/s), *z* is the valence of the ion, *F* is Faraday’s constant (96,488.46 C/mol), *R* is the gas constant (8.3143 J/(mol·K)), *T* is the temperature in Kelvin (K), *φ* represents the potential of the external electric field (V), and *x* denotes the distance of the ion from the entry edge within the material (mm).

Luo et al. [74] conducted research on the degradation processes of cementitious materials under the coupled action of electric pulses and sulfate. The transport process of SO_4_^2−^ in cement mortar specimens with and without mineral admixtures under the coupling of the two and the deterioration of the mortar were investigated experimentally. The results demonstrated that electric pulses significantly accelerated the migration of external SO_4_^2−^ into the specimens, leading to a rapid increase in the internal concentration of SO_4_^2−^ and thus accelerating the rate of degradation of the cement mortar due to sulfate attack [73]. Incorporation of mineral admixtures was found to enhance the durability of the cement mortar. However, there is still a lack of research on whether the external electric field affects the chemical reaction processes of sulfate attack, and studies on the transport model of SO_4_^2−^ in cementitious materials under the coupled action of electric fields and sulfates are yet to be conducted.

## 4. Influence of Internal Environmental Factors on the Sulfate Attack Resistance of Concrete

### 4.1. Internal Sources of Corrosion

During construction, raw materials, soil, groundwater, river water, or seawater can introduce sulfates into concrete structures, which become internal sources of corrosion once the concrete has hardened. The phenomenon of internal sulfate attack (ISA) was first identified in the mid-1980s in prestressed concrete railroad ties and has since become increasingly common in underground structures and marine engineering projects [76]. Internal sulfate attack involves a chemical reaction between the sulfates released during the oxidation of pyrite and the aluminates in Portland cement, resulting in the formation of expansive secondary ettringite. This formation of ettringite leads to dimensional changes, increased internal stresses, and cracking in the concrete, thus affecting the structure’s performance and durability. The diffusion of sulfates within concrete is driven by concentration gradients between the concrete and its surrounding environment. The concentration of sulfates in concrete not only depends on the external supply and accumulation, but also changes as the sulfates are consumed in corrosion reactions within the concrete.

When concrete lacks internal sources of corrosion, SO_4_^2−^ ions penetrate the surface and diffuse into the interior over time. Consequently, the concentration of sulfates near the exposed surface of the specimen is relatively high. Corrosion reactions occur, forming corrosion products that gradually fill the initial cracks in the outer layers of the concrete. Once these initial cracks are excessively filled with degradation products, the expansion of these cracks and the formation of new cracks are observed. These newly expanded or formed cracks provide additional physical pathways for the diffusion of sulfate solutions, significantly increasing the rate of sulfate diffusion within the concrete. Therefore, in concrete without internal sources of corrosion, the accumulation of sulfates is initially controlled by the concentration gradient. In the later stages, the accumulation of sulfates is driven both by the concentration gradient and the direct diffusion paths provided by the crack system.

**Figure 9:**
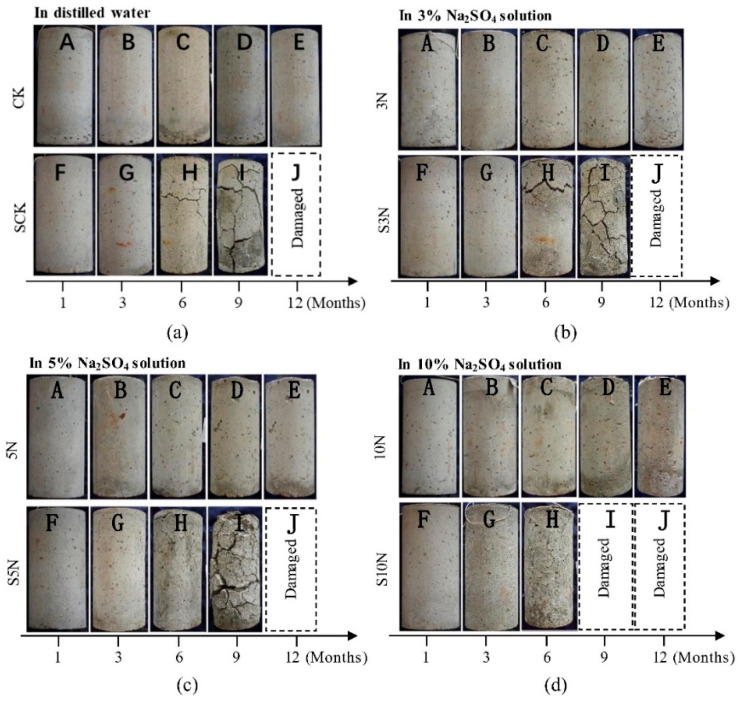
Effect of internal corrosion sources on sulfate-corroded concrete [77]. Changes of specimen appearance immersed in: (**a**) distilled water, (**b**) 3% Na_2_SO_4_ solution, (**c**) 5% Na_2_SO_4_ solution, and (**d**) 10% Na_2_SO_4_ solution. (note: CK specimens are not premixed sulfates. SC specimens are premixed sulfates. Corrosion type of 3N, 5N and 10N specimens is external sulfate corrosion (ESC). Corrosion type of SCK specimens is internal sulfate corrosion (ISC). Corrosion type of S3N, S5N and S10N specimens is internal sulfate corrosion (ISC). internal-external combined sulfate corrosion (CSC)).

Concrete with internal corrosion sources exhibits more pronounced deterioration and earlier structural failure in varying concentrations of sulfate solutions, as illustrated in Figure 9 [77]. This increased susceptibility to damage arises because internal corrosion sources generate a higher volume of degradation products in the initial phases. These products accumulate and become embedded within the concrete, thereby obstructing strength development and inducing the formation of initial cracks. During the initial stages, sulfates are driven by the concentration gradient potential to either exit or permeate the concrete [78]. This process facilitates the formation of additional cracks, which eventually merge to form a comprehensive network of interconnected cracks, thus providing enhanced physical pathways for the infiltration of the sulfate solution [76]. Consequently, the sulfate solution penetrates the concrete directly through this network, causing substantial damage. Therefore, in concrete with internal corrosion sources, the direct infiltration of sulfate solution through the crack network emerges as the predominant mechanism of sulfate transport in later stages [79].

### 4.2. Cement Chemical Composition

**Figure 10:**
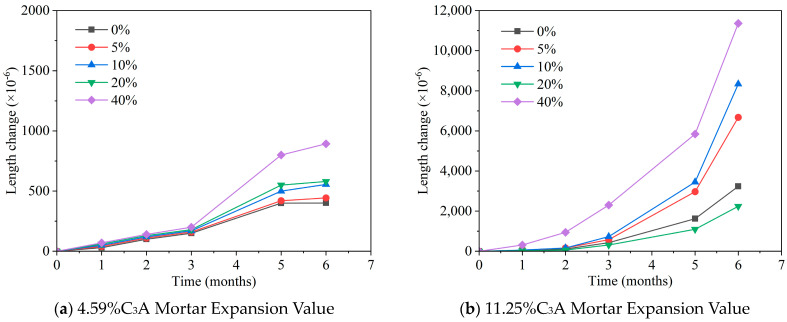
Effect of C_3_A content on the sulfate attack resistance of mortar [81] (note: the different colored lines in the graph represent different percentages of limestone).

Since 1930, the resistance of Portland cement to sulfate attack has been closely associated with its tricalcium aluminate (C_3_A) content. Over the decades, international cement standards such as ASTM, AFNOR, BS, and DIN have acknowledged the critical influence of aluminium oxide, leading to the implementation of limitations on its presence in sulfate-resistant Portland cement [80]. Tosun [81] conducted experiments by preparing mortars with cements containing 4.59% and 11.25% C_3_A. Additionally, 5%, 10%, 20%, and 40% limestone powder were added to each of the two types of cement, and their expansion was measured after six months. The results demonstrated that higher C_3_A content leads to more significant mortar expansion, as shown in Figure 10, directly affecting the durability of the cement mortar against sulfate attack. However, the C_3_A content alone does not fully dictate the performance of cement in such conditions. Cements with identical C_3_A levels can exhibit varying degradation rates, influenced by factors such as the clinker’s production process and the crystalline structure of the C_3_A.

In addition to sulfate resistance, high C_3_A content can negatively affect the demand for high-efficiency water reducers and workability in fresh concrete due to its high initial adsorption capacity. Conversely, the hydration products formed by the reaction of tetracalcium aluminoferrite (C_4_AF) with water exhibit better sulfate resistance than those of C_3_A. Research indicates that a high C_4_AF content enhances the sulfate resistance of Portland cement [82,83], with C_4_AF content in sulfate-resistant cements ranging from 16–18% [84,85,86].

The cementitious materials incorporating limestone filler are more susceptible to a special type of sulfate corrosion, known as thaumasite corrosion, due to the presence of carbonate ions, as shown in Equation (9). This deterioration can proceed faster in cold environments (below 15 °C), and it is particularly more deleterious when sulfates are associated with a low pH, as both contribute to the decomposition of C–S–H, as shown in Equation (10).

(9) $$3{Ca}^{2+}+{SiO}_{3}^{2-}+{CO}_{3}^{2-}+{SO}_{4}^{2-}+15H_{2}O\to 3CaO\cdot {SiO}_{2}\cdot {CO}_{2}\cdot {SO}_{3}\cdot 15H_{2}O$$

Thaumasite

(10) $$3{Ca}_{3}{Si}_{2}O_{7}\cdot 3H_{2}O+xH_{2}O\to 2({SiO}_{2}\cdot \frac{x}{2}H_{2}O+3Ca{\left(OH\right)}_{2}$$

Decalcification of CSH

American standards suggest that the contents of C_3_A and 2C_3_A + C_4_AF in sulfate-resistant cement should be limited to 5% and 25%, respectively [81]. Comparative studies on different cement types have shown that the resistance to the thaumasite form of sulfate attack (TSA) diminishes in the sequence of composite cement (PAC) made with a 1:1 mass ratio of ordinary portland cement (OPC) to sulfate aluminate cement (SAC), followed by SAC, sulfate-resistant cement (SRC), and OPC. Thus, the hierarchy of cement performance against TSA is PAC > SAC > SRC > OPC.

Tricalcium silicate (C_3_S) is the component with the highest content in Portland cement, so its content is also considered to be an important parameter affecting the sulfate resistance of Portland cement [87]. An increase in the content of C_3_S implies an increase in the release of calcium hydroxide (CH) during the hydration process of Portland cement, thereby altering the development of the strength, heat, and porosity of the hardened paste. Since a significant amount of CH precipitated from the hydration of C_3_S is a necessary reactive phase for gypsum formation, reducing the content of C_3_S can correspondingly reduce the generation of ettringite and gypsum, thereby enhancing the sulfate resistance of concrete [88].

### 4.3. Water-Cement Ratio

It has been suggested by [60] that the refinement of the internal pore size of concrete exacerbates the damage caused by internal crystallisation, and thus concrete with lower water–cement ratios should be more susceptible to physical damage than those with higher water–cement ratios. Firstly, the higher permeability of concrete systems ensures that a larger volume of concrete is affected by the ingress of sulfates, leading to expansion. Additionally, in concrete systems with a low water–cement ratio, the initially low porosity may result in precipitated ettringite and gypsum clogging further pathways for sulfate ingress. However, due to the high pore connectivity in high water–cement ratio concrete, pore pressure is transmitted over longer distances through the liquid phase, ultimately leading to a combined effect in various pores that results in greater expansion.

**Figure 11:**
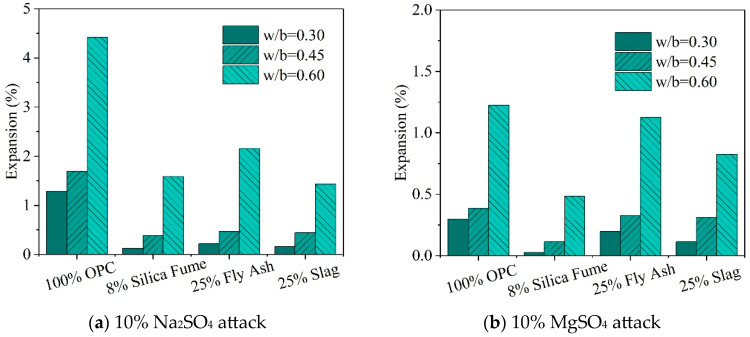
Effect of different water–cement ratios on the expansion of polycellular cemented concrete under sulfate attack [35].

Conversely, Nehdi et al. [35] found that higher water–cement ratios correlate with increased surface degradation and expansion rates. Figure 11 illustrates the effect of different water–cement ratios on the expansion of polycellular cemented concrete under sulfate attack. Their observations indicated that the increase in expansion rate from a water–cement ratio of 0.45 to 0.6 is notably more pronounced than from 0.3 to 0.45. They argued that well-compacted and cured low water–cement ratio concrete exhibits improved compactness, which diminishes permeability and thus expansion. Since high water–cement ratios generally result in larger pores, it is commonly believed that the expansion stress caused by crystal growth is less pronounced in these larger pores [15]. Nevertheless, the explanation for the observed higher expansion in high water–cement ratio concrete remains unaddressed. The reduction in the water–cement ratio effectively decreases the depth of Na_2_SO_4_ crystal damage from the concrete surface to its interior [89], enhancing its resistance to sulfate attack.

**Figure 12:**
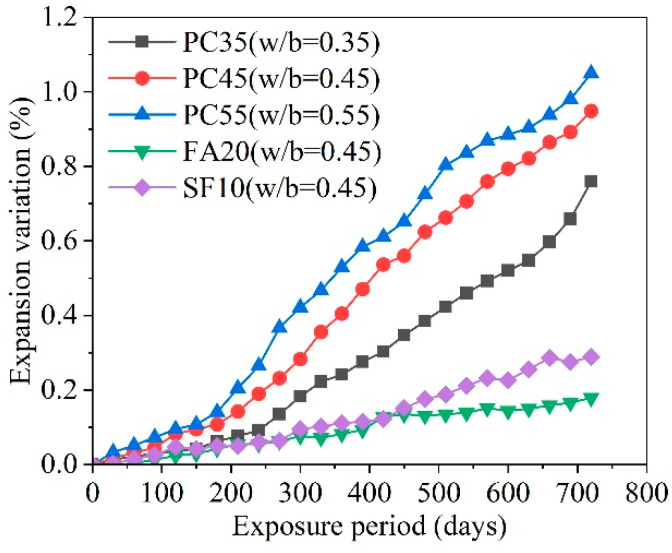
Development of expansion rate of concrete with different water–cement ratios in 2.1% Na_2_SO_4_ solution [90] (note: PC represents ordinary Portland cement, FA20 indicates that 20% of the cement is replaced with fly ash, and SF10 indicates that 10% of the cement is replaced with silica fume).

Research by Zhang et al. [90] indicates that the expansion rate of concrete escalates with the water–cement ratio, documenting expansion rates of 0.76%, 0.95%, and 1.05% for concrete at water–cement ratios of 0.35, 0.45, and 0.55, respectively, after 720 days under sulfate attack. Figure 12 presents the development of the expansion rate of concrete with different water–cement ratios in 2.1% Na_2_SO_4_ solution. Liu et al. [91] observed that concrete at different water–cement ratios maintained relatively intact states in the evaporation zone during sulfate exposure. With increasing water–cement ratios, the capillary suction initially decreased and then increased, and the height of the evaporation zone first increased and then decreased. This indicates that a water–cement ratio of 0.45 provides better resistance to corrosion for the evaporation zone compared to ratios of 0.35 and 0.55, resulting in the lowest amount of sulfate ingress into the evaporation zone.

## 5. Methods to Improve Corrosion Resistance

Currently, there are two main approaches to reducing sulfate attack on reinforced concrete: first, by enhancing the performance of the concrete matrix itself, increasing the density and hydrophobicity of the concrete, which inhibits the transmission of sulfates into the concrete matrix; and second, by applying a coating treatment to the concrete surface, isolating the concrete matrix from corrosive media.

### 5.1. Mineral Admixture

Since Ca(OH)_2_ and calcium aluminate hydrate are involved in the sulfate attack reaction, any measure that can limit the presence of these products is expected to improve resistance to sulfate attack. The partial replacement of cement with SCM is beneficial in improving the resistance of concrete to sulfate attack because the volcanic ash reaction of the supplementary cementitious material (SCM) can consume Ca(OH)_2_ in the concrete matrix. Additionally, replacing OPC cement with SCM effectively reduces the content of C_3_A in cementitious materials. Many studies have claimed that when SCM (e.g., low-calcium fly ash [92,93], finely ground blast furnace slag powder [94], metakaolin [95], and silica fume [42]) partially replaces cementitious materials, sulfate resistance can be improved. Al-Amoudi [1] suggested that SCMs enhance the sulfate resistance of concrete through three primary mechanisms. The first one is that the addition of SCM alters the clinker composition and dilutes its content, leading to a reduction in C_3_A levels. This change reduces the formation of aluminate hydrates in the system that can react with external sulfates. The second concludes that as the hydration reaction progresses, SCMs continue to consume Ca(OH)_2_ due to their pozzolanic activity, leading to the production of C-S-H gels. This reaction reduces the availability of Ca(OH)_2_ to react with external sulfates to form gypsum or to leach calcium, which could contribute to the formation of additional ettringite. The final mechanism posits that hardened concrete containing SCMs exhibits an improved pore structure due to enhanced particle packing and pozzolanic reactions. This results in a denser microstructure, which diminishes susceptibility to external sulfate penetration, thereby enhancing the concrete’s durability.

Low-calcium fly ash typically exhibits higher resistance to sulfate attack compared to high-calcium fly ash. This is because low-calcium fly ash can consume more Ca(OH)_2_ produced by the hydration reactions of cement, forming more sulfate-resistant C-S-H gels and C-A-S-H gels. It does not contribute additional reactive phases present in high-calcium fly ash, which can accelerate sulfate-induced degradation. In contrast, high-calcium fly ash may produce additional Ca(OH)_2_ during hydration, thereby accelerating the degradation induced by sulfates [96]. In addition to the difference in calcium content, the proportions of oxides in fly ash (including silicon dioxide, aluminum oxide, and iron oxide, as well as their amorphous and crystalline phases) significantly affect its resistance to sulfate attack. It is speculated that fly ash with less than 5% CaO content and without reactive aluminum oxide does not react with external sulfates to form expansive ettringite [97]. An R factor has been proposed to predict the sulfate resistance of fly ash mixtures, considering the content of CaO and Fe_2_O_3_ in the fly ash. The R factor is calculated using Equation (11):

(11) $$R=\frac{CaO\left(\%\right)-5}{{Fe}_{2}O_{3}}\%$$

The enhancement of sulfate resistance in slag–cement blends is commonly attributed to the consumption of silicates, pore refinement, and a reduction in permeability. Furthermore, controlling the C_3_A content in cement can also regulate its performance, which is dependent on the cement’s composition and the extent of slag replacement. The higher aluminum oxide content in slag can produce C-S-H gels with a lower Ca/Si ratio, which ultimately forms hydrotalcite by incorporating aluminum [98]. This process limits the reaction of free aluminum within the system with external sulfates, thereby enhancing sulfate resistance [99,100]. Research by Cao et al. [101] indicates that optimal improvement in expansion and strength characteristics occurs when the slag content ranges between 50–70%, particularly when the aluminum oxide content is 14.25% or 16.25%, respectively. Thus, employing slag with a higher aluminum oxide content as a partial replacement for cement is a favorable strategy for mitigating sulfate attack.

### 5.2. Surface Coating

Compared to modifying the concrete matrix, surface coating protection provides the advantages of simple application and cost-effectiveness. Concrete surface coatings can be divided into organic and inorganic types, each capable of forming a dense film on the surface to varying extents. This film acts to fill and compact the capillary pores on the concrete surface. Inorganic cementitious protective coatings are noted for their excellent weather resistance and anti-aging properties. High-quality organic film coatings are characterized by their low porosity and low water permeability, which effectively prevent the penetration of external salt solutions into the concrete structure. Moreover, these coatings offer excellent breathability, thus preventing the formation of blisters on the concrete surface layer when exposed to rising temperatures [102].

Huang et al. [103] experimented by mixing an acrylic polymer emulsion with ordinary Portland cement in a specific ratio and applying it to the concrete surface, effectively reducing the penetration of sulfate solutions into the concrete. Nia et al. [104] performed accelerated sulfate tests and found that the service life of polyurethane and epoxy resin coatings increased by approximately 14.5 years and 11 years. Suleiman et al. [105] assessed various surface treatment methods for their resistance to corrosion, noting that asphalt coatings offered protection comparable to epoxy resins. However, when asphalt is applied to high-porosity concrete at an early stage, water molecules may become trapped between the asphalt coating and the concrete, thereby increasing the likelihood of delamination. In contrast, epoxy coatings, which exhibit higher adhesion [106], typically do not encounter this issue.

### 5.3. Nanomaterials

**Figure 13:**
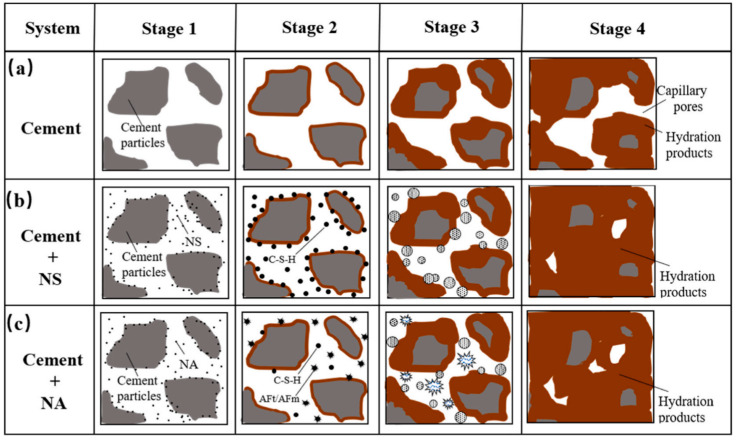
Schematic diagram of the hydration progress of (**a**) pure cement, (**b**) cement with NS (nano-SiO_2_), and (**c**) cement with NA (nano-Al_2_O_3_) [108].

Nanoparticles, due to their large specific surface area, participate to varying degrees in the hydration reaction of cement, which can enhance the density of concrete and thus improve its resistance to sulfate attack. Extensive research has been conducted on the impact of various nanoparticles, such as micro silica [107], nano silica [108], nano-TiO_2_ [109], nano-Al_2_O_3_ [108], and nano-CaCO_3_ [110], on the sulfate resistance of concrete. Nano silica, in particular, has garnered significant attention due to its unsaturated oxygen and silicon bonds, which facilitate chemical reactions with Ca(OH)_2_, forming a C-S-H nucleus. Additionally, nano silica exhibits high reactivity, initially existing in the form of H_2_SiO_4_^2−^, which then reacts with Ca^2+^ ions released from hydration products to form additional C-S-H gels, as represented in Equation (12). This gel fills the voids between cement particles, enhancing concrete density as depicted in Figure 13b. Xu et al. [109] explored the effects of nano-TiO_2_ modification on concrete’s sulfate resistance, finding that nano-TiO_2_ reduces the mass loss rate of concrete and enhances its sulfate resistance. Furthermore, research by Lu et al. [111] demonstrated that nano silica provides a superior filling effect compared to nano-TiO_2_.



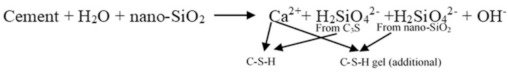

(12)


The application of nano-Al_2_O_3_ involves the presence of amphoteric aluminium in the form of Al(OH)^4−^ in the highly alkaline pore solution, as shown in Equation (13) [108].

Subsequently, the dissolved Al(OH)^4−^ reacts with gypsum in the cement paste to form ettringite, as shown in Equation (14), which further promotes the hydration of C_3_A, as illustrated in the second phase of Figure 13c. This process reduces the number of large pores within the cement matrix and interrupts the connected capillary channels, thereby hindering the diffusion of ions within the sample and the evaporation of water, enhancing the resistance of the cementitious material to Na_2_SO_4_ attack. Compared to other nanomaterials, nano-CaCO_3_ is relatively inexpensive and more readily obtained from materials such as limestone and marble. It accelerates the formation of carbonate aluminate complexes from tricalcium aluminate (C_3_A) and also reacts with tricalcium silicate (C_3_S) to hasten the setting and early strength development. Studies have shown [110] that incorporating 1% nano-CaCO_3_ can enhance the resistance of concrete to sulfate attack and significantly increase the service life of the concrete.

(13) $${Al}_{2}O_{3}\cdot xH_{2}O+2{OH}^{-}\to Al(OH{)}_{4}^{-}+(x-3)H_{2}O$$

(14) $$2Al(OH{)}_{4}^{-}+6{Ca}^{2+}+3{SO}_{4}^{2-}+4{OH}^{-}+26H_{2}O\;\;\;\;\;\;\;\;\;\;\to 3CaO\cdot {Al}_{2}O_{3}\cdot 3{CaSO}_{4}\cdot 32H_{2}O$$

### 5.4. Layered Double Hydroxides (LDHs)

**Figure 14:**
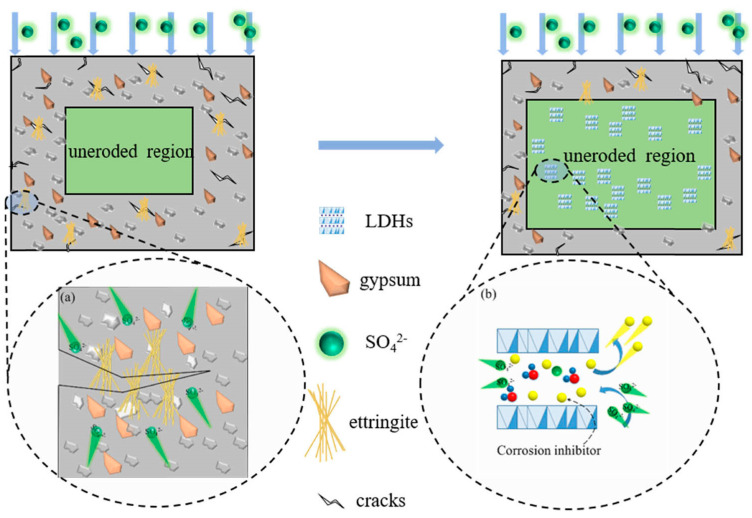
Schematic illustration of sulfate attack-induced crack and the modification of LDHs in concrete: (**a**) the schematic illustration of crack induced by sulfate attack; (**b**) modification mechanism of LDH on sulfate [113].

Layered double hydroxide (LDH) materials, due to their structural characteristics, play significant roles in various fields such as environmental engineering, medicine, the chemical industry, and agriculture, although their application in concrete engineering is still relatively limited. The use of LDHs in concrete materials originated from the development of organic–inorganic composite water-reducing agents. Some researchers have also explored the adsorption characteristics of LDHs materials for common corrosive ions in concrete, such as CO_3_^2−^, Cl^−^, and SO_4_^2−^. Figure 14 illustrates the sulfate attack-induced crack and the modification of LDHs in concrete.

Li et al. [112] conducted research on the adsorption of SO_4_^2−^ by calcined LDH materials, confirming that the products of calcined LDHs exhibit good adsorption capacity for SO_4_^2−^ in environments with low pH values. The mechanisms for the adsorption of SO_4_^2−^ by LDHs and their calcined counterparts are identified as “ion exchange” and “memory effect”, respectively [113], as shown in Figure 11. Parker et al. [114] contend that high-valence anions are more readily exchanged into the interlayers of LDHs, while low-valence anions are more likely to be exchanged out. Furthermore, the uniform structure and extensive internal surface area of LDHs facilitate the accommodation of guest molecules. For corrosive ions commonly found in concrete, such as Cl^−^ and SO_4_^2−^, certain anions exhibit lower exchange capacities and reduced stability, rendering them effective adsorbents for SO_4_^2−^ in concrete. This insight offers researchers a method to modify concrete using LDH materials to exchange and adsorb Cl^−^ and SO_4_^2−^ in the pore solution of cement concrete.

Zhai et al. [113] proposed the direct incorporation of LDH materials into concrete to leverage their adsorptive properties. Tatematsu [115] employed the interlayer anion exchange capabilities of hydrotalcite to remove Cl^−^ and SO_4_^2−^ from concrete. Tsujimura’s research [116] validated the efficacy of LDHs in adsorbing Cl^−^ and SO_4_^2−^, noting that the removal rate of SO_4_^2−^ in solutions is generally independent of the solution’s temperature and the initial concentration of SO_4_^2−^. Research by Guimarães et al. [117] has shown that under specific conditions, the removal rate of anions by LDHs can reach up to 90%. Guo [118] conducted a systematic study on the application of Mg-Al-CO_3_ type LDH materials in mortar, revealing that in the alkaline system formed by cement hydration products, LDH materials have a certain adsorption capacity for corrosive anions. However, while LDH materials influence the flowability of mortar, they do not significantly impact its compressive strength.

### 5.5. Geopolymers

**Figure 15:**
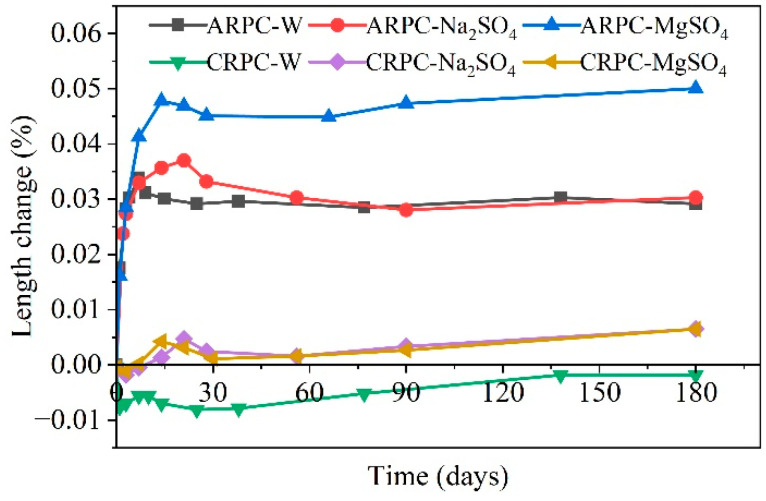
Patterns of changes in ARPC and CRPC lengths as affected by sulfate corrosion [123] (note: CRPC represents conventional Portland cement-based reactive powder concrete, and ARPC represents alkali-activated slag-based reactive powder concrete).

Due to the more compact microstructure of geopolymers compared to cementitious materials, and their superior pore structure and more stable hydration products [119], geopolymers exhibit notably higher resistance to salt corrosion than Portland cement [120]. Further research indicates that geopolymer resistance to corrosion from MgSO_4_ and MgCl_2_ solutions is slightly lower than its resistance to Na_2_SO_4_ and NaCl_2_ solutions [121], while its resistance to sulfate attack is marginally less than its resistance to chloride solution corrosion [122]. Researchers investigated the dimensional changes in conventional Portland cement-based (CRPC) and alkali-activated slag-based geopolymer concrete (ARPC) specimens after six months of immersion in water, 10% Na_2_SO_4_ solution, and 10% MgSO_4_ solution. The results showed that the length change of geopolymer concrete specimens immersed in Na_2_SO_4_ solution was nearly identical to those immersed in water, whereas immersion in MgSO_4_ solution led to a 66% increase in expansion [123], as depicted in Figure 15. After three months of immersion in a 10% Na_2_SO_4_ solution, the loss rates in compressive strength and flexural strength of geopolymer concrete were 1.0% and 9.0%, respectively, while after three months in a 10% MgSO_4_ solution, the losses were 3.0% and 16.0%, respectively.

Özdal et al. [124] evaluated the effects 5% Na_2_SO_4_, 5% MgSO_4_, 5% NaCl, and 5% MgCl_2_ solutions on the mass and compressive strength of slag-based geopolymer concrete. It was observed that after exposure to these salt solutions, the mass change in the geopolymer concrete was negligible, at about 0.1%, but there was a substantial reduction in strength, with sulfate solutions having a more aggressive corrosive impact than chloride. After 12 months of exposure to the solutions, the strength loss rates in geopolymer concrete were 21.6%, 16.4%, 3.6%, and 4.7%, respectively, for 5% Na_2_SO_4_, 5% MgSO_4_, 5% NaCl, and 5% MgCl_2_ solutions.

The deterioration of compressive strength in geopolymer concrete due to sulfate attack is principally linked to the increase in matrix porosity and the development of cracks. Increasing the concentration of Na_2_SO_4_ solution from 5% to 20% led to an increase in the 28-day strength loss rate from 12.6% to 19.5%. This finding suggests that higher concentrations of sulfate solutions intensify the formation of cracks and pores in geopolymer concrete, significantly impairing its mechanical properties [125].

## 6. Conclusions and Future Outlook

This paper systematically reviews the deterioration patterns and damage mechanisms of concrete under sulfate attack, considering both external environmental factors and intrinsic material properties. The conclusions drawn from the synthesis and summary of existing research on the resistance of concrete to sulfate attack are as follows, with prospects for future research outlined:

(1) Currently, the mechanisms causing performance degradation in concrete due to sulfate attack are primarily divided into physical and chemical damage. Theories supporting physical damage suggest that the precipitation of sulfate crystals or volumetric expansion create pressure within the concrete, leading to deterioration of concrete properties. In contrast, chemical damage theories posit that SO4^2−^ ions penetrate the concrete’s pores, participating in hydration reactions and forming expansive products like ettringite and gypsum, which cause cracking. In practical engineering, the durability of cement-based materials is usually controlled by multiple factors; therefore, a deeper understanding of the coupling mechanisms between various factors under composite corrosion is essential.
(2) Numerous environmental factors significantly influence the resistance of concrete structures to sulfate attack, and can even alter the mechanisms of sulfate attack in concrete. The effect of ambient temperature change on the production of expansion in sulfate damaged concrete shows a two-phase pattern. Firstly, variations in environmental humidity, sulfate solution concentration, and type can change the mechanisms and reaction products of sulfate attack in concrete, leading to different types of damage. Then, cycles of wetting-drying and freezing-thawing both accelerate the rate of sulfate attack in concrete, significantly speeding up performance deterioration. Moreover, the presence of chlorides can impact concrete deterioration to varying degrees. The presence of stray current will significantly accelerate the transmission of SO_4_^2−^, which will have a negative impact on the performance of concrete.
(3) The characteristics of concrete materials to some extent determine their resistance to sulfate attack. Internal sources of corrosion can lead to the early production of more degradation products, and the accumulation and embedding of these products can inhibit strength development and produce more cracks. Adjusting the content of various components in cement clinker or adding supplementary cementitious materials can enhance the sulfate resistance of concrete. At present, the effect of water-cement ratio on sulfate-corroded concrete is controversial. As geopolymer has a denser pore structure and more stable hydration products, these advantages result in a significantly higher salt corrosion resistance of geopolymer concrete than silicate cement concrete. There is a lack of research on the sulfate resistance of low-carbon cementitious materials such as magnesium-based cements (e.g., potassium magnesium phosphate cement), improved silicate cements (e.g., C_3_S_2_ cementitious materials), and limestone-calcined clay cements (LC^3^ cements), and the research and development of high-corrosion-resistant low-carbon cementitious materials is of great significance to the promotion of the sustainable development of the cement industry.
(4) At present, relatively effective methods for improving the sulfate resistance of concrete that have attracted more attention from scholars mainly include the use of supplementary cementitious materials (SCM), nanomaterials, layered double hydroxides (LDHs), and coatings on concrete. There are three different mechanisms for improving the sulfate resistance of concrete by adding SCM. In addition, the type and proportion of auxiliary cementing materials will also affect the modification effect. The two modification methods, nanomaterials addition and concrete surface coating, mainly fill the internal pores of concrete and prevent the transport of aggressive ions to achieve the purpose of improving the performance of concrete. The modification mechanism of layered double hydroxides (LDHs) is to adsorb the aggressive ions inside the concrete. LDHs have a better modification effect, while SCM has the advantage that the material is easier to obtain and the price is lower.

However, in practical engineering, the durability of cement-based materials is typically influenced by multiple factors. Therefore, it is crucial to thoroughly understand the coupling mechanisms between these factors under combined corrosion conditions. This is a pressing issue that requires urgent attention. In addition to providing a literature review, our future research will focus on collecting and analyzing extensive experimental data to establish more comprehensive and accurate models that elucidate the combined effects of various factors on the sulfate resistance of concrete. By applying data fitting and multivariable analysis techniques, it will be possible to quantitatively assess the impact of different influencing factors. For instance, regression analysis and machine learning algorithms can be utilized to develop models that predict the deterioration rate and damage extent of concrete under complex environmental conditions. Through in-depth analysis and extrapolation of these multiple factors, researchers can propose more scientific and practical protective measures and design guidelines. This approach will enhance the durability of concrete structures in complex environments and provide a robust theoretical foundation and data support for future engineering practices.

Additionally, there is a notable lack of research on the sulfate resistance of new low-carbon cementitious materials, such as magnesium-based cements (e.g., potassium magnesium phosphate cement), enhanced Portland cements (e.g., C_3_S_2_ cementitious materials), and limestone calcined clay cement (LC^3^). Developing highly corrosion-resistant low-carbon cementitious materials is important for promoting the sustainable development of the cement industry.

## Figures and Tables

**Table 1:** Effect of solution concentration on sulfate attack of concrete.

Ref.	Concentration of Sulfate Solution	Corrosion Age	Conclusion
Ou et al. [38]	3%, 5%, and 7% Na_2_SO_4_ solution	14 d, 28 d, 55 d, 83 d, 110 d	Concrete resistance to sulfate attack increases progressively in damage with increasing sodium sulfate concentration.
Fang et al. [33]	15% and saturated Na_2_SO_4_ solution; 13% and saturated MgSO_4_ solution	0 d, 28 d, 56 d, 84 d, 154 d	When the concentration of sodium sulfate solution is less than 15%, the higher the concentration, the faster the degradation rate of the flexural and compressive resistance coefficients of the specimens. Specimens in a 15% sodium sulfate solution exhibit the fastest rate of deterioration in their resistance coefficients. However, the rate of decline in the flexural and compressive resistance coefficients of specimens immersed in a saturated sodium sulfate solution is slower compared to those in a 15% sodium sulfate solution. When the concentration of magnesium sulfate solution is below 13%, an increase in concentration leads to a faster degradation rate in both the flexural and compressive resistance coefficients of the specimens. Specimens in a 13% magnesium sulfate solution experience the most rapid decline in their resistance coefficients. However, the rate of decline in the flexural and compressive resistance coefficients of specimens in a saturated magnesium sulfate solution is slower than that in a 13% magnesium sulfate solution.
Fan et al. [39]	0%, 10%, 15%, and 20% Na_2_SO_4_ solution	210 d	As sulfate concentrations increase, the peak stress in concrete decreases, and the surface exhibits an increased density of pores. Concurrently, the size and number of internal pores within the concrete expand, providing a more direct route for sulfate ion infiltration. This process facilitates the accumulation and expansion of corrosive products, which compromises the internal structure of the concrete. Consequently, there is a macroscopic reduction in both the peak stress and the elastic modulus, effects that become increasingly pronounced with higher sulfate concentrations. The concrete specimens demonstrate shear failure, marked by a diagonal crack that extends through the entire sample. As the concentration of corrosion increases, the severity of the material’s crushing also intensifies.
Chen [40]	0%, 3%, 4%, 5%, and 6% Na_2_SO_4_ solution	24 d, 45 d, 60 d	Cement with a high water–cement ratio produces more ettringite when immersed in a solution with sulfate ion concentrations of 3–4%. Conversely, cement with a low water–cement ratio generates a greater amount of ettringite at a sulfate ion concentration of 6%. High-concentration sulfate solutions continuously influence the formation of ettringite in cement with a high water–cement ratio and accelerate the formation of ettringite in cement with a low water–cement ratio.

## Data Availability

No new data were created or analyzed in this study.
